# Estimating healthcare costs of acute gastroenteritis and human campylobacteriosis in Switzerland

**DOI:** 10.1017/S0950268816001618

**Published:** 2016-08-12

**Authors:** C. SCHMUTZ, D. MÄUSEZAHL, P. J. BLESS, C. HATZ, M. SCHWENKGLENKS, D. URBINELLO

**Affiliations:** 1 Swiss Tropical and Public Health Institute, Basel, Switzerland; 2 University of Basel, Basel, Switzerland; 3 Epidemiology, Biostatistics and Prevention Institute, University of Zurich, Zurich, Switzerland; 4 Institute of Pharmaceutical Medicine, University of Basel, Basel, Switzerland

**Keywords:** Acute gastroenteritis, campylobacteriosis, healthcare costs, Switzerland

## Abstract

Rising numbers of campylobacteriosis case notifications in Switzerland resulted in an increased attention to acute gastroenteritis (AG) in general. Patients with a laboratory-confirmed *Campylobacter* infection perceive their disease as severe and around 15% of these patients are hospitalized. This study aimed at estimating healthcare costs due to AG and campylobacteriosis in Switzerland. We used official health statistics, data from different studies and expert opinion for estimating individual treatment costs for patients with different illness severity and for extrapolating overall costs due to AG and campylobacteriosis. We estimated that total Swiss healthcare costs resulting from these diseases amount to €29–45 million annually. Data suggest that patients with AG consulting a physician without a stool diagnostic test account for €9·0–24·2 million, patients with a negative stool test result for *Campylobacter* spp. for €12·3 million, patients testing positive for *Campylobacter* spp. for €1·8 million and hospitalized campylobacteriosis patients for €6·5 million/year. Healthcare costs of campylobacteriosis are high and most likely increasing in Switzerland considering that campylobacteriosis case notifications steadily increased in the past decade. Costs and potential cost savings for the healthcare system should be considered when designing sectorial and cross-sectorial interventions to reduce the burden of human campylobacteriosis in Switzerland.

## INTRODUCTION

Since 1995 *Campylobacter* spp. has been the most frequently reported gastrointestinal bacterial pathogen in humans in Switzerland [1] and since 2005 in the European Union (EU) [2]. An estimated 9·25 million cases of campylobacteriosis occurred in 2009 in the 27 EU member states, of which around 2% were reported [3]. Havelaar *et al.* estimated the ‘true’ incidence rate of campylobacteriosis in these countries at 30–13 500/100 000 population (350/100 000 in Switzerland).

In Switzerland, positive test results for *Campylobacter* spp. have to be notified by diagnostic laboratories to the Federal Office of Public Health (FOPH) since 1988 [4]. In 2012, 8480 campylobacteriosis cases were registered within the National Notification System for Infectious Diseases (NNSID), which is the highest number reported so far [1]. This corresponds to a notification rate of 105 cases/100 000 resident population in Switzerland. The extent to which campylobacteriosis contributes to the public health burden of acute gastrointestinal illness is unknown. In The Netherlands, about twice the population size of Switzerland, approximately 4·8 million cases of gastroenteritis occur annually, whereby 220 000 patients need medical consultation [5].

A study among 69 general practitioners (GPs) concluded a rising awareness of campylobacteriosis as a public health problem in Switzerland (Supplementary material). Despite its mostly self-limiting nature, the health burden of campylobacteriosis in the Swiss population may be significantly higher than figures from the NNSID indicate. Severe cases and complications such as Guillain–Barré syndrome, reactive arthritis and post-infectious irritable bowel syndrome amplify the burden of disease and in particular the economic burden [6–8].

The estimated economic burden (equating healthcare costs at large, including, e.g. loss of productivity and/or transportation and other direct and indirect non-healthcare costs) of gastrointestinal infections or foodborne illnesses in high-income countries varies between €14 (Australia [9]) and €1305 (United States [10]) per case in the community ([9–20] in Table 1). Thereby, healthcare costs account for €3–155/case in the community [9–20]. This wide range is partially due to heterogeneity in case definitions and definitions of economic burden. The yearly costs for gastroenteritis due to 14 food-related pathogens and associated sequelae in The Netherlands were estimated at around €468 million [11].

**Table 1.** Overview of selected studies estimating the cost of illness of gastrointestinal or foodborne illnesses

First author, year [ref.]	Nation	Year	Pathogens/disease considered (community cases, unless specified otherwise)	Cases per year	Costs included^a^				Exchange rate used (€1 = …)^c^	Direct healthcare cost, per case (in €)	Direct healthcare cost, yearly (in million €)	Total costs per case (in €)	Total yearly costs (in million €)
					Direct healthcare cost	Patient costs (e.g. travel costs)	Productivity losses	Others^b^					
Hoffmann, 2015 [10]	United States	2013	15 foodborne pathogens including long-term disabilities; only domestically acquired and foodborne cases	8 914 713	X		X	X	USD 1·34	155^d^	1384	1305^d^	11 636
Mangen, 2015 [11]	Netherlands	2011	14 foodborne pathogens; including sequelae	4810000	X	X	X		EUR 1	31^d^	147	97^d^	468
Scharff, 2012 [12]	United States	2010	All domestically acquired, foodborne illnesses	47 780 778	X		X	X	USD 1·33	75	3568^d^	806–1227	38 506–58 589
Friesema, 2012 [13]	Netherlands	2009	Gastroenteritis	4 600 000	X	X	X		EUR 1	14^d^–32^d^	63–147	133–151	611–695
Gauci, 2007 [14]	Malta	2004/05	Infectious intestinal disease	164 471	X	X	X		Lm 0·44^e^	72^d^	12	108	17
Abelson, 2006 [15]	Australia	2004	Gastroenteritis due to foodborne illnesses	5 400 000^f^	X		X	X	AUD 1·69	22^d^	118	111^d^	598
Majowicz, 2006 [16]	City of Hamilton, Canada	2001	Acute gastroenteritis	619 334^g^	X		X		CAD 1·39	17^d^	11^d^	66	40
Van den Brandhof, 2004 [17]	Netherlands	1999	Gastroenteritis	4 476 399	X	X	X		EUR 1	14	61	77	345
Roberts, 2003 [18]	England	1994	Infectious intestinal disease	9 400 000^h^	X	X	X		GBP 0·66 (year 1999)	16^d^–44^d^	153–412	109^d^–120	1028–1128
Hellard, 2003 [9]	Australia	1999	Highly credible gastroenteritis	15 173 430	X		X		AUD 1·65	3^d^	46	14^d^	208
Lindqvist, 2001 [19]	Municipality of Uppsala, Sweden	1999	Foodborne illnesses	500 000^i^ (Sweden)	X		X		SEK 8·81	117	58^d^	246	123
Scott, 2000 [20]	New Zealand	1999	Foodborne infectious disease	119 320	X	X	X	X	NZD 2·01	9^d^	1·0	229	27
Karve, 2014 [21]	United States	2010/11	Acute gastroenteritis; only cases consulting a physician, visiting emergency department and inpatient care setting	6 668 944^j^	X				USD 1·36	472^d^	3151	472	3151

a Categories represent only a very broad classification of costs included in the studies. Certain items may be included in different categories, depending on the study. For example, transportation cost was sometimes considered as ‘direct healthcare cost’ (when covered by the health system) and sometimes included in ‘patient costs’.

b For example, food recalls, or intangible costs for reduced quality of life (intangible costs are monetary representations of pain, suffering and fear which can be obtained through willingness-to-pay studies [22]), or value of statistical life for premature deaths.

c Average exchange rates of the calendar year when the study was conducted (as indicated in the column ‘year’) were used and extracted from [23].

d Calculated based on yearly case numbers and either costs per case (for calculating yearly costs) or yearly costs (for calculating costs per case) as reported in the original publication.

e Exchange rate as indicated in the original publication (1 Maltese lira = €2·29).

f According to Hall *et al.* 2005 [24].

g Calculated based on a population size of 490 290 and 126 320 cases/100 000 population as reported in the original publication.

h Calculated based on total yearly costs (£742·8 million) divided by total costs per case (£79) as reported in the original publication, rounded to the next 100 000.

i According to Norling, 1994 [25].

j Sum of estimated annual episodes of acute gastroenteritis in physician's office (5 337 473), emergency department (1 032 064) and inpatients (447 580) as reported in the original publication.

For campylobacteriosis, the estimated economic burden per case varies, ranging from €117 (The Netherlands [17]) to €6141 (United States [12]) ([8, 10–12, 17, 20, 26] in Table 2). Healthcare costs of campylobacteriosis cases were estimated at €8/case in New Zealand, €82–280 in The Netherlands and €163–253 in the United States ([8, 10–12, 20] in Table 2). These numbers are difficult to compare as case definitions and cost items included vary between studies. For example, sequelae due to campylobacteriosis were considered in some studies while in others they were not. Campylobacteriosis-associated acute gastroenteritis (AG) accounts for approximately 108 000 cases/year in The Netherlands, causing annual societal costs of about €81·5 million (including sequelae) [11]. In the EU, campylobacteriosis cases account for expenditures of public health systems and for productivity losses of around €2·4 billion/year according to the European Food Safety Authority [28]. The economic burden highlights the importance of this widespread and common disease.

**Table 2.** Overview of selected studies estimating the cost of illness of campylobacteriosis

First author, year [ref.]	Nation	Year	Pathogens/disease considered (community cases, unless specified otherwise)	Sequelae considered	Cases per year	Costs included^a^				Exchange rate used (€1 = …)^c^	Direct healthcare cost, per case (in €)	Direct healthcare cost, yearly (in million €)	Total costs per case (in €)	Total yearly costs (in million €)
						Direct healthcare cost	Patient costs (e.g. travel costs)	Productivity losses	Others^b^					
Hoffmann, 2015 [10]	United States	2013	*Campylobacter* spp.; only domestically acquired and foodborne cases	GBS	845 024^f^	X		X	X	USD 1·34	253^d^	213	1710	1445
Mangen, 2015 [11]	Netherlands	2011	*Campylobacter* spp.	GBS, ReA, IBS, IBD	108 000	X	X	X		EUR 1	280^d^	30	757	82
Scharff, 2012 [12]	United States	2010	*Campylobacter* spp.; only domestically acquired and foodborne cases	GBS, ReA	845 024^e^	X		X	X	USD 1·33	163	138^d^	1392–6141	1177–5189
Gellynck, 2008 [26]	Belgium	2004	*Campylobacter*-associated gastroenteritis and sequelae	GBS, ReA, IBD	55 000	X	X	X		EUR 1	n.a.	n.a.	497^d^	27
Mangen, 2005 [8]	Netherlands	2000	*Campylobacter* spp. and sequelae	GBS, ReA, IBD	79 000	X	X	X		EUR 1	82^d^	6·5	261^d^	21
Van den Brandhof, 2004 [17]	Netherlands	1999	*Campylobacter* spp.	Not considered	79 000^f^	X	X	X		EUR 1	n.a.	n.a.	117^d^	9
Scott, 2000 [20]	New Zealand	1999	Proportion of foodborne *Campylobacter* spp.	GBS, ReA, HUS	75 345	X	X	X	X	NZD 2·01	8^d^	0·6	265	20
Roberts, 2003 [18]	England	1994	*Campylobacter* spp.	Not considered	n.a.	X	X	X		GBP 0·66 (year 1999)	n.a.	15	n.a.	106

GBS, Guillain–Barré Syndrome; HUS, haemolytic uraemic syndrome; IBD, inflammatory bowel disease; IBS, irritable bowel syndrome; n.a., not available; ReA, reactive arthritis.

a Categories represent only a very broad classification of costs included in the studies. Certain items may be included in different categories, depending on the study. For example, transportation cost was sometimes considered as ‘direct healthcare cost’ (when covered by the health system) and sometimes included in ‘patient costs’.

b For example, food recalls, or intangible costs for reduced quality of life (intangible costs are monetary representations of pain, suffering and fear which can be obtained through willingness-to-pay studies [22]), or value of statistical life for premature deaths.

c Average exchange rates of the calendar year when the study was conducted (as indicated in the column ‘year’) were used and extracted from [23].

d Calculated based on yearly case numbers and either costs per case (for calculating yearly costs) or yearly costs (for calculating costs per case) as reported in the original publication.

e According to Scallan *et al.* 2011 [27].

f Assumed, according to Mangen *et al.* 2005 [8].

A quantification of healthcare costs due to AG and/or campylobacteriosis in Switzerland is lacking so far. Due to the rising number of campylobacteriosis case notifications in recent years, we conducted several studies which aimed at generating a better understanding of the epidemiology of campylobacteriosis in Switzerland. We investigated epidemiological determinants [29], described time trends in notification data [1], the campylobacteriosis-associated illness experience from the patients' perspective [29, 30], the case management strategies of GPs (Supplementary material) and laboratory positivity rates of *Campylobacter* spp. (Supplementary material). In concert, these studies indicate that campylobacteriosis is causing a considerable burden of disease which considerably impacts the health system in Switzerland and is likely associated with high costs.

The aim of this study was to estimate the total annual costs for the medical treatment of campylobacteriosis in Switzerland. However, given that available data do not systematically distinguish campylobacteriosis from AG we focused this analysis on available data of both conditions. To the best of our knowledge, this is the first study estimating healthcare costs due to AG and campylobacteriosis in Switzerland.

## METHODS

We developed patient management models and estimated their frequency and associated costs from the perspective of the healthcare system.

### Typology of patients: patient management models

Cost estimation was based on four different patient management models for AG which were derived from a broad expert consultation across a purposive enquiry among practitioners in private general and specialized practices (four), clinics and university hospitals (four), authors opinions and data available to them: (i) patients consulting a physician without stool testing (patient management model A), (ii) patients consulting a physician with negative *Campylobacter* stool test results (patient management model B), (iii) patients consulting a physician and having a positive *Campylobacter* stool test result (patient management model C), and (iv) hospitalized campylobacteriosis cases (patient management model D).

### Population figures as basis for modelling: sources and approach

The number of notified campylobacteriosis cases occurring each year in Switzerland was retrieved from the NNSID [1]. A study assessing the trend in *Campylobacter* positivity rates was conducted (thereafter referred to as the ‘Positivity study’). This study used data of eight Swiss diagnostic laboratories on *Campylobacter* tests performed between 2003 and 2012. Positivity rates, defined as the proportion of *Campylobacter*-positive to total number of *Campylobacter* tests, were calculated. The number of *Campylobacter* tests performed in Switzerland was estimated based on the preliminary positivity rate of 2012.

In 2013, a qualitative study among 69 GPs was conducted in Switzerland (thereafter referred to as the ‘Swiss GP study’). Using a semi-structured questionnaire, physicians were interviewed about their case management strategies for and general perception of AG and campylobacteriosis. From this study, GPs' estimates on the proportion of AG patients with a stool test prescribed were available.

In 2014, the Swiss Sentinel Surveillance Network decided to study AG for 12 months; 170 participating GPs reported all cases consulting due to AG. This study (thereafter referred to as the ‘*Sentinella* study’) also provides estimates on the proportion of patients with a stool test.

The results used for cost estimates from the ‘Positivity’, the ‘Swiss GP’ and the ‘*Sentinella* study’ are preliminary. Short summaries of these studies including the preliminary results used for estimating healthcare costs can be found in the Supplementary material. Final results of all these studies will be published separately.

We used the number of hospitalizations due to the ICD-10 code ‘A04·5 *Campylobacter* enteritis’ as reported in official hospital statistics published by the Federal Statistical Office [31]. We compared this number with estimates based on the hospitalization rate found in our case-control study on determinants of campylobacteriosis [29] and the number of campylobacteriosis case notifications from the NNSID [1].

#### Population-level estimates

The number of campylobacteriosis cases registered at the FOPH was assumed to correspond to the number of patients in management models C and D in the whole of Switzerland. The number of hospitalizations in Switzerland (patient management model D) was extracted from official hospital statistics (hospitalizations due to *Campylobacter* enteritis, ICD-10 code A04·5) [31]. 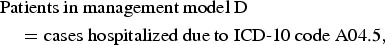


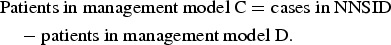


The proportion of positive to total number of campylobacteriosis tests was used to estimate the number of patients in management model B based on notified cases (hence, cases with a positive test result). 
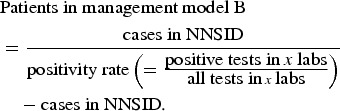


The proportion of patients with stool testing (as opposed to consultation without stool testing) was used to estimate case numbers for patient management model A. 
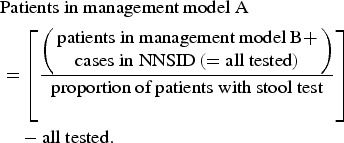


**Fig. 1.**
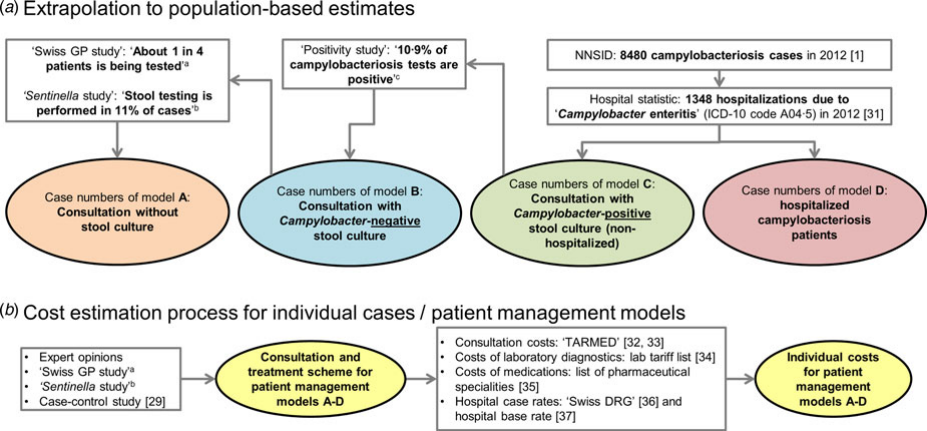
Overview of data sources used for (*a*) extrapolation of treatment costs and (*b*) for cost estimation for acute gastroenteritis and campylobacteriosis patients. ^a^ Qualitative study about case management of campylobacteriosis patients among 69 general practitioners in Switzerland (Supplementary material). ^b^ Study on acute gastroenteritis conducted within the Swiss Sentinel Surveillance Network ‘*Sentinella*’ (www.sentinella.ch) in 2014 (Supplementary material). ^c^ Study on laboratory positivity rates of *Campylobacter, Salmonella* and *Shigella* diagnostic tests in Switzerland (Supplementary material).

The data sources used for the extrapolation from individual to population-based costs are summarized in Figure 1*a*.

### Healthcare expenditures

Healthcare costs for each of the patient management models were estimated by combining associated medical standard procedures with publicly available respective rates for accounting. We extrapolated these individual case management costs to estimate healthcare costs associated with AG and campylobacteriosis in Switzerland in 2012.

#### Sources of cost data

We used different sources in order to calculate healthcare expenditure due to *Campylobacter* infections: from the Swiss GP study, based on expert opinions and using preliminary results of the *Sentinella* study, treatment schemes and standard approaches for case management (including number and duration of consultations, laboratory tests performed and medications prescribed) were identified. Consultation costs of GPs were calculated using the number of points from the publicly available Swiss medical tariff system, TARMED (as of June 2012) [32] and a point value of €0·7138 which is used in the canton of Bern [33]. Similarly, points for laboratory diagnostics were extracted from the official tariff list (‘Analysenliste’; as of January 2012) using a point value of €0·83 applied throughout Switzerland [34]. Costs for medications were extracted from the list of pharmaceutical specialities (‘Spezialitätenliste’, version of 1 January 2012) [35]. Calculation of hospitalization costs was based on the flat rates of the Swiss diagnosis-related group-based (DRG-based) hospital reimbursement system and a base rate which is applied by several regional hospitals in the canton of Bern, both for 2012 [36, 37]. Costs in Swiss francs were converted to Euros using an exchange rate for the Euro of €0·83 per Swiss franc (average exchange rate January 2012–December 2012) [23]. The cost estimation process for the patient management models is presented in Figure 1*b*.

We obtained primary cost data from invoices for consultations of *Campylobacter*-positive patients, covering all patient consultations between 2011 and 2013 at the Swiss TPH travel clinic. This part of the study was approved by the local ethical committee (Ethikkommission Nordwest- und Zentralschweiz ref. no. EKNZ: 2014–159).

### Data analysis

#### Costs per patient treated

Differentiating by patient management model (models A–D), we evaluated the costs for consultations, medication, laboratory tests and hospitalization until conclusion of medical treatment. For all patient management models we defined two scenarios to account for some of the heterogeneity of the patients and the case management strategies within a given model: a minimal and an extended or prolonged scenario. The proportions of patients treated with the minimal and the extended scenario were estimated based on results of the case-control (e.g. proportion of patients treated with antibiotics) [29] and the *Sentinella* study (e.g. number of consultations; Supplementary material). Afterwards, experts were asked whether they considered the estimated proportions reasonable. The two scenarios do not imply any chronology of the steps involved.

Estimates for patient management model C were validated using real patient records of the Swiss TPH travel clinic. Patient invoices were entered in an electronic database and analysed using Stata v. 13 (StataCorp., USA). Costs for laboratory tests or medication not primarily associated with AG were excluded, i.e. tests for *Echinococcus, Filaria*, flavivirus and *Plasmodium*, vaccines for rabies and tetanus, and electrocardiograms. Laboratory tests performed in external laboratories were invoiced by these laboratories and could, hence, not be considered in our analysis. However, we added costs for one positive stool test for *Campylobacter* spp. as patients were selected based on having laboratory-confirmed campylobacteriosis.

## RESULTS

### Frequency of different patient management models in Switzerland

In the NNSID, 8480 cases of campylobacteriosis were registered in 2012 [1]. Preliminary results from the Positivity study showed that 10·9% of all campylobacteriosis tests were positive (Supplementary material). Consequently, we estimated that 77 798 tests for *Campylobacter* spp. were made in 2012, of which 69 318 had a negative test result (patient management model B). Estimates of the Swiss GP study indicated that one in four AG patients has a stool test performed (Supplementary material), suggesting that 233 394 patients consult a physician each year without further stool testing (patient management model A). However, preliminary results from the *Sentinella* study suggest that only 11% (420/3794) of patients had stool testing performed (Supplementary material). In this case a total of 629 457 patients would be in patient management model A.

**Fig. 2.**
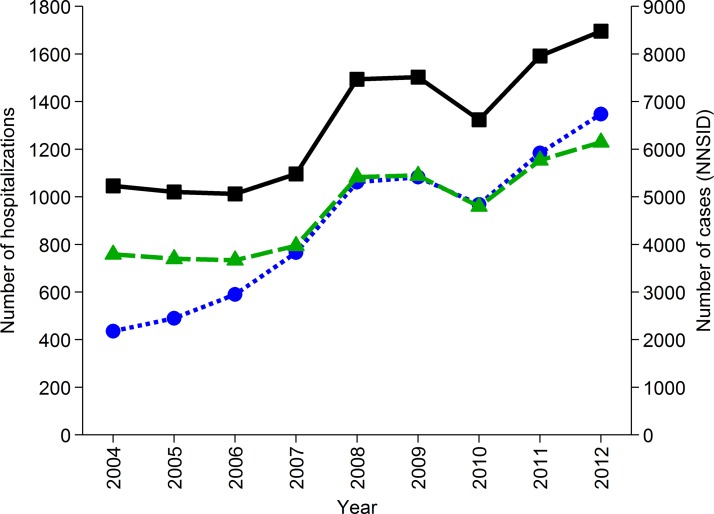
Number of hospitalizations due to ICD-10 code A04·5 ‘*Campylobacter* enteritis’ in Switzerland from 2004 to 2012 (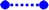, left axis, [31]), number of hospitalizations extrapolated from results of a case-control study in Switzerland [29] assuming hospitalization of 14·5% of cases registered in the National Notification System for Infectious Diseases (NNSID) (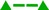, left axis) and number of case notifications from the NNSID [1] (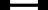; right axis).

The number of hospitalizations due to ‘*Campylobacter* enteritis’ (ICD-10 code A04·5) as reported in the official Swiss hospital statistics increased steadily since 2004. In 2012, 1348 hospitalizations were reported which is the maximum so far (Fig. 2). For comparison, 14·5% (23/159) of interviewed patients in the recent case-control study, with laboratory-confirmed campylobacteriosis, reported hospitalization due to their illness [29]. Considering the case notification numbers of 2012 (8480 cases), this proportion would result in 1230 hospitalizations (patient management model D). Patient management model C includes all notified cases except those being hospitalized (1348), resulting in 7132 patients annually in Switzerland.

### Individual case management costs for AG and campylobacteriosis patients

The costs per case are highly variable ranging from €30 (patient management model A) to €4828 (patient management model D). The cost items attributed to the different patient management models and scenarios and associated costs are presented in Table 3. (For a list of unit costs see Supplementary Table S2.)

**Table 3.** Healthcare costs associated with the management of acute gastroenteritis and campylobacteriosis for four patient management models with two scenarios each (values reflect costs in €)

	Patient management model A Consultation without stool test		Patient management model B Consultation with negative stool culture^a^		Patient management model C Consultation with positive stool culture^a^		Patient management model D Hospitalization	
Minimal scenario	10 min consultation^b^	19·02	15 min consultation^b^	31·69	15 min consultation^b^	31·69	15 min consultation^b^	31·69
	1 medication^c^	10·79	Stool culture^a^ (negative)	64·74	Stool culture^a^ (positive)	128·65	Hospital stay (DRG G67B^e^)	4727·36
			Taking blood sample	5·85	Taking blood sample	5·85		
			Haemogram^d^ and CRP	18·26	Haemogram^d^ and CRP	18·26		
			1 medication^c^	10·79	1 medication^c^	10·79		
			5 min reviewing patient file	12·68	5 min reviewing patient file	12·68		
			5 min telephone cons.	12·68	5 min telephone consultation	12·68		
Total, minimal scenario		**29·81**		**156·68**		**220·59**		**4759·06**
Extended scenario	+ Taking blood sample	5·85	+ Antibiotic	24·90	+ Antibiotic	24·90	+ 5 min reviewing patient file	12·68
(costs additional to minimal scenario)	+ Haemogram^d^ and CRP	18·26	+ Pharmacy fees^f^	6·27	+ Pharmacy fees^f^	6·27	+ Taking blood sample	5·85
	+10 min second consultation^b^	19·02	+10 min second consultation^b^	19·02	+10 min second consultation^b^	19·02	+ Haemogram^d^ and CRP	18·26
							+ 15 min second consultation^b^	31·69
Total, extended scenario		**72·93**		**206·87**		**270·78**		**4827·53**
Proportion of patients requiring extended scenario:	**20%**		**40%**		**65%**		**50%**	
Data sources								
Expert opinion	x		x		x		x	
TARMED^g^ [32]	x		x		x		x	
List of pharmaceutical specialities [35]	x		x		x		x	
Official laboratory tariff list [34]	x		x		x		x	
Flat rates of Swiss DRG, version 1.0 [36]							x	
Swiss GP study^h^	x		x		x			
*Sentinella* study^i^	x		x		x			
Swiss TPH travel clinic					x			

CRP, C-reactive protein; NNSID, National Notification System for Infectious Diseases.

a Stool culture includes *Campylobacter, Salmonella* and *Shigella.*

b Or telephone consultation of same duration.

c Of the following medications: antidiarrhoeal, antiemetics, probiotics; average price of those medications: €10·79 (13 CHF).

d Including erythrocytes, leucocytes, haemoglobin, haematocrit, thrombocytes, and ⩾5 subpopulations of leucocytes.

e For a patient with *Campylobacter* enteritis (ICD-10 code A04·5), aged ⩾1 year, with a length of stay between 2 and 11 nights, the DRG group ‘G67B’ is assigned. Cost weight: 0·573, base rate (applied by several regional hospitals in Bern): €8250·20 (9940 CHF) [37]. Quote from Swiss DRG version 1.0 [36] defining code ‘G67B’: [*translated from German*] ‘Oesophagitis, gastroenteritis and other diseases of the digestive organs with a complex diagnosis or age <1 year or gastrointestinal bleeding, with very severe or severe complications or comorbidities or age >74 years or peptic ulcer disease with severe complications or comorbidities or age >74 year, hospital occupancy > 1 day, without complicating diagnosis, without dialysis’.

f Fees include check of the prescription which can be invoiced once per item prescribed (‘Medikamenten-Check’; €3·57, CHF 4·30) and check of the purchase which can be invoiced once per patient, per day and per provider (‘Bezugs-Check’; €2·70, CHF 3·25) [49].

g Costs vary among cantons; median costs are used (tariff point value €0·7138 or 0·86 CHF, e.g. canton Bern) [33].

h Qualitative study about case management of campylobacteriosis patients among 69 general practitioners in Switzerland (Supplementary material).

i Study on acute gastroenteritis conducted within the Swiss Sentinel Surveillance Network ‘*Sentinella*’ (www.sentinella.ch) in 2014 (Supplementary material).

The healthcare costs of 41 patients with laboratory-confirmed *Campylobacter* spp. infection were analysed. Costs for those 19 male and 22 female patients aged between 1 and 72 years were in the range of €179–1033 (median €464). The number of consultations varied between 1 and 8 per patient (median 2), the number of blood samples taken between 0 and 4 (median 1) and the time between the first and the last consultation between 0 (only one consultation) and 65 days (median 3). Consultation costs and costs for laboratory testing of real patient data were higher than estimated costs for patient management model C (Supplementary Table S3).

### Healthcare costs due to AG and campylobacteriosis

Total healthcare costs for the management of the four different patient management models combined in Switzerland in 2012 were estimated at €29·5–44·7 million (Table 4). Costs for the different patient management model groups (A–D) were €9·0–24·2, €12·3, €1·8 and €6·5 million, respectively (Supplementary Fig. S1).

**Table 4.** Estimated healthcare costs for the treatment of acute gastroenteritis and campylobacteriosis in Switzerland. Costs for individual cases are based on resource use estimates presented in Table 3

	Patient management model A_Sentinella_	Patient management model A_Swiss GP_	Patient management model B	Patient management model C	Patient management model D
Estimated number of cases (*n*)	**629 457**	**233 394**	**69 318**	**7132**	**1348**
In minimal scenario	503 566	186 715	41 591	2496	674
In extended scenario	125 891	46 679	27 727	4636	674
Consultation	€11 969 523	€4 438 134	€4 359 611	€448 552	€42 722
Laboratory diagnostics	€0	€0	€5 753 394	€1 047 762	€0
Medication	€6 791 841	€2 518 321	€747 941	€76 954	€0
Hospitalization	€0	€0	€0	€0	€6 372 487
+Consultation	€3 129 858	€1 160 517	€527 246	€88 156	€33 845
+Laboratory diagnostics	€2 298 770	€852 359	€0	€0	€12 307
+Medication	€0	€0	€864 154	€144 488	€0
Healthcare costs by patient management model	**€24 189 992** ^a^	**€8 969 331** ^a^	**€12 252 346** ^a^	**€1 805 913** ^a^	**€6 461 362** ^a^
Total healthcare costs	**€29 488 953–44 709 613^a^**				

a Totals do not always add up because of rounding.

Costs separated by type/provider were: €11·1–20·6 million for GPs' services (including medical assistants), €7·7–9·1 million for laboratory diagnostics, €4·4–8·6 million for medications and €6·4 million for hospitalizations (Supplementary Fig. S2).

## DISCUSSION

This study provides for the first time an assessment of total Swiss healthcare costs due to AG and campylobacteriosis by estimating the individual costs of four types of patient management models and their frequency: patients suffering from AG and seeking medical care without being tested (model A); patients seeking medical care and having a *Campylobacter*-negative stool test (model B); patients seeking medical care and having a *Campylobacter*-positive stool test (model C); and patients with a severe course of campylobacteriosis requiring hospitalization (model D).

Cases of campylobacteriosis increased in the last decade 1·5-fold, implying a contemporarily relevant public health problem. We estimated that in Switzerland, each year 311 192–707 255 patients consult a physician due to AG or campylobacteriosis, leading to annual healthcare costs ranging from €29 to €45 million.

The calculations were based on several assumptions as this study provides the first estimates of healthcare costs due to AG and campylobacteriosis in Switzerland. The country has no central database which is based on diagnostic codes and where healthcare costs from outpatient care are systematically recorded. Therefore, we tried to cross-validate our estimates whenever possible by combining different data sources. The real patient data which we used for comparison with cost estimates for patient management model C originated from our own institution's (Swiss TPH) travel clinic. These real patient data suggested higher costs for laboratory-confirmed, ambulatory patients than we used for our calculations. Possibly consultation time in returning travellers was longer because of the travel anamnesis and laboratory tests were more extensive. Nevertheless, returning travellers are likely to be overrepresented also in the patients with AG seen by GPs. When using the median total costs of the real patient data of the travel clinic for patients in management model C, the costs for this group would be €3·3 million (instead of €1·8 million; Supplementary Figs S1 and S2). Hence, we believe the cost estimates used for patient management model C are conservative.

Some physicians reported performing a second stool test after a positive result for certain patient groups (e.g. working in the food sector) before allowing the patients to return to work. A few experts claimed that the consultation times we applied in our models were rather short. They suggested consultation times of 5–10 min longer for selected (but not for all) consultations. The case-control study [29] found that about 10% of campylobacteriosis patients in outpatient treatment received intravenous therapy, which was not considered in our models. Furthermore, patients requiring hospitalization may be transferred to the hospital by ambulance causing additional costs. Taking all these points into account, we believe that our estimates reflect rather conservative approximations.

### Healthcare costs of laboratory-confirmed campylobacteriosis patients

Campylobacteriosis cases as registered in the NNSID were estimated to cost around €8·3 million/year (patient management models C and D). The majority of these costs are attributable to hospitalizations. Comparison of our estimates with actual patient data suggests that our estimates (at least for patient management model C) underestimated actual costs occurring in the health system. The number of hospitalizations due to ‘*Campylobacter* enteritis’ (ICD-10 code A04·5) matches well with the calculated number of hospitalized patients using the official notification data together with the hospitalization rate found in the case-control study (1348 *vs.* 1230 cases). The hospitalization costs, which are based on DRG flat rates, include all costs occurring during the hospital stay. This flat rate is independent of the length of stay as long as it is within 2–11 nights (for DRG code G67B, according to DRG v. 1.0 [36]).

### Healthcare costs of AG patients

The costs for AG patients without laboratory-confirmed campylobacteriosis varied significantly depending on the proportion of stool testing we used to calculate patient numbers for patient management model A. The proportion of stool testing is highly variable also in other countries: it was found to be 12% in The Netherlands [38], 19–44% in the United States [39, 40] and 27% in England [41]. Even though our estimate of 11% from the *Sentinella* study is lower compared to the proportions reported in other countries we believe that this number is more accurate than the semi-quantitative estimates obtained from the Swiss GP study. Moreover, the figure from the *Sentinella* study represents the proportion of patients for which the physician initiated stool testing. It is likely that not all patients actually provided a stool specimen. Hence, using the proportion of actually completed stool tests would increase case numbers in model A and our cost estimates. Additionally, our calculation for patient management model A is based on the estimated number of tests for *Campylobacter* spp. This may in fact underestimate the total number of stool tests as in some instances physicians might only test their patients for viruses, for example. In this case, the number of patients in management models A and B would be even larger.

Apart from *Campylobacter* both *Salmonella* and *Shigella* infections are notifiable in Switzerland. Usually, basic stool bacteriology involves testing for these three pathogens [42]. Under this assumption and ignoring the chance of mixed infections, all *Salmonella*- or *Shigella*-positive patients were assigned to management model B (patients with *Campylobacter*-negative stool test). This leads again to a rather conservative estimate of costs since stool cultures with a positive result are more expensive than negative stool cultures (€64·74 *vs*. €128·65) [34]. Additionally, salmonellosis and shigellosis patients may also need hospitalization and those patients are, therefore, more likely to create costs similar to those estimated for campylobacteriosis patient management models C and D. In 2012, 1243 cases of salmonellosis and 159 cases of shigellosis were reported [43, 44]. Moreover, AG patients with viral infections and patients without an identified causative agent might be hospitalized. The hospitalization costs for these patients were not considered in our study.

Patients consulting a physician not at all or only by phone and patients seeking help in a pharmacy have not been considered in this study. Up to 60% of gastroenteritis patients calling the medical practice are managed by phone, according to the Swiss GP study (Supplementary material). Individual (healthcare) costs for these patients may be low. However, the high quantity of these patients might still lead to considerable costs.

### Comparison of cost estimates for Switzerland with estimates of other countries

Various studies have been conducted in several countries to estimate costs for gastrointestinal infections or campylobacteriosis (Tables 1 and 2). However, comparison of costs is very difficult due to varying case definitions used, heterogeneity in costs included, differences in health systems and health-system use and time. We estimated that a case of laboratory-confirmed campylobacteriosis costs on average €975 (average per case for models C and D). The extent of underreported campylobacteriosis infections – defined as infections in individuals who seek healthcare but whose infection is not captured by the surveillance system [45] – is unknown for Switzerland. The multiplication factor due to underreporting of campylobacteriosis was estimated at 1·3 in the UK [46] and at 2·0–5·6 in The Netherlands [6, 47]. Applying the same factors to Swiss data would result in 2544– 39 008 additional campylobacteriosis cases. Assuming that underreporting was due to under-diagnosis (as opposed to under-notification), these cases are automatically included in our patient management model A (where model A represents all consulting AG patient without stool diagnostics.) Hence, costs in model A attributable to under-diagnosed campylobacteriosis cases would range between €0·98 and €1·50 million. Total costs attributable to campylobacteriosis would then range between €8·4 and €9·8 million in Switzerland (representing 19–33% of total AG costs) or €206–759/case. Healthcare costs per case are higher than Dutch (€82–280/case, Table 2) or US estimates (€163–253/case). However, the latter two were based on the yearly estimated number of campylobacteriosis cases in the population while we considered only campylobacteriosis cases presenting to the GP or being hospitalized.

On average, a case of AG (including campylobacteriosis) in Switzerland was estimated at €63–95. Again, our cost estimates are based on cases presenting to the GP while estimates from other countries usually are presented for cases in the community. Hence, values are not comparable even though our cost estimates are within the range of cost estimates from other countries (€3–155 [9–20], Table 1).

### Unknown socioeconomic burden

We only assessed direct healthcare costs for AG and campylobacteriosis. The average hospital stay of three nights and the median disease duration of 7 days of campylobacteriosis patients which were found in the case-control study [29] suggest that the socioeconomic burden due to productivity loss and home care is a multiple of the healthcare costs. Additionally, we neither considered costs arising from complications of the disease (e.g. Guillain–Barré syndrome, reactive arthritis or irritable bowel syndrome) nor did we include out-of-pocket expenses for medications of patients not consulting a physician or costs arising of patients consulting the physician exclusively by phone. This further underscores the conservative nature of our overall healthcare cost estimated at €29–45 million.

The disease burden and economic consequences are further increased by years of life lost due to premature mortality. The ICD-10 codes A02 ‘other *Salmonella* infections’ and A04·5 ‘*Campylobacter* enteritis’ were recorded only for four patients in 2011 as the main cause of death (Swiss Federal Statistical Office, personal communication). When considering also secondary causes of deaths, 104 deaths were registered in 2011. For influenza it was shown that mortality is underreported in official statistics [48]. We assume that such underreporting is also the case for deaths due to campylobacteriosis (and salmonellosis).

AG and campylobacteriosis cause a marked public health problem generating considerable costs. To our knowledge, this is the first study investigating healthcare costs due to AG and campylobacteriosis in Switzerland. Further research is needed for more accurate cost estimation. In order to reduce the financial burden and suffering of patients, there is a need for implementing health policy measures, sectorial and inter-sectorial public health interventions and increasing awareness in the population at all levels.

## Supplementary material

For supplementary material accompanying this paper visit http://dx.doi.org/10.1017/S0950268816001618. click here to view supplementary material
