# Supplementary material for: Estimating healthcare costs of acute gastroenteritis and human campylobacteriosis in Switzerland
Source: Epidemiol Infect. 2016 Aug 12;145(4):627–41. doi: 10.1017/S0950268816001618 (PMC5426335; doi:10.1017/S0950268816001618)
Supplement: Supplementary file 1 [file S0950268816001618sup001.docx]

*Epidemiology and Infection*

Estimating health care costs of acute gastroenteritis and human campylobacteriosis in Switzerland

C. Schmutz, D. Mäusezahl, P. J. Bless, C. Hatz, M. Schwenkglenks, D. Urbinello

Supplementary material

**“Swiss GP study”**

**Summary of a qualitative study among 69 general practitioners in Switzerland (manuscript under review)**

**Introduction**

In Switzerland, the number of campylobacteriosis cases reported to the National Notification System for Infectious Diseases (NNSID) is increasing [1]. Interpretation of these surveillance data is challenging as the patients’ health care seeking behaviour and the physicians’ diagnostic and treatment behaviour are unknown.

**Methods**

In 2013, Swiss general practitioners were interviewed using a semi-structured questionnaire. They were asked about their perception of acute gastroenteritis and campylobacteriosis in daily practice and about their strategies in managing patients with these diseases (focussing on diagnostic and treatment approaches). Interviews were analysed using inductive content analysis.

**Selected preliminary results**

Physicians agreed that campylobacteriosis has been more frequent in recent years.

Interviewer: “*How do you experience diarrhoeal diseases, campylobacter in particular, among your patients? Is it a big problem?*”

Physician PH09: “*Yes. I also observed that the number of cases [campylobacter] is increasing, especially around New Year, I have the impression, I had a few cases, but always sporadic ones.*”

Some physicians estimated how frequently they order stool testing in a patient with diarrhoea:

Interviewer: “*How many stool bacteriologies do you do… if you say among 10 patients with acute diarrhoea*”

Physician PH22: “*Well, I would think about one of them […].*”

Interviewer: “*If you estimate, roughly, how many patients do you test, out of 10?*”

Physician SF01: “I think I test 3… 3-4.”

On average, physicians estimated to order a stool diagnostic in around 1 in 4 patients presenting with acute diarrhoea.

Not all patients calling the primary care practice because of acute gastroenteritis will finally come for a consultation. While physicians largely agreed on this general statement, their estimates on the proportion of patients managed by phone were highly variable:

Interviewer: “*If you estimate, what percentage of the ones that call you for diarrhoea do finally come for a consultation?*”

Physician PH09: “Mostly it is also the wish of the patient to come. And probably it is around 40% of patients that finally come. At first, I wanted to say fifty-fifty, but maybe 40% will come. […]”

Interviewer: “*Are there as well patients who just contact you by phone and do not come to the practice?*”

Physician PH07: “*This exists exceptionally. Someone calls, says he has diarrhoea and that he needs something […]. This exists, indeed, but they [medical assistants] point out that they [patients] have to come to the practice in case of fever.*”

Interviewer: “*You say it is a minority who calls and does not come for consultation. How many would you guess do that, maybe 10%?*”

Physician PH07: “*No, fewer. This is really an exception.*”

**“Positivity study”**

**Summary of a study on time trends in positivity rates of Campylobacter spp. in Switzerland**

**Introduction**

Campylobacteriosis is the most frequent bacterial, foodborne infection reported to the National Notification System for Infectious Diseases (NNSID) in Switzerland [1]. Case numbers have been increasing since the beginning of reporting in 1988. It is unknown whether this increase is due to a change in disease incidence or whether it could be explained by other factors such as increased testing frequency. This study looked at time trends in the proportion of positive campylobacter tests out of all campylobacter tests performed (=positivity rate).

**Methods**

A total of 11 diagnostic laboratories were selected from a list of laboratories contributing most campylobacteriosis case notifications to the NNSID and invited to participate in the study. Eight laboratories agreed to participate and provided patient-related data (sex, age, canton of residence, patient identification code assigned by laboratory) and test-related data (test result, date of test, type of test) for all campylobacter tests performed between 2003 and 2012. Positivity rates were defined as:

$$Positivity rate \left( in \% \right)= \frac{Total number of tests positive for campylobacter}{Total number of tests performed for campylobacter}*100$$

**Selected preliminary results**

Participating laboratories provided data of 194 487 campylobacter tests conducted between 2003 and 2012. Some laboratories could not provide data over the whole study period. Data were only included if laboratories provided data for a complete calendar year. After exclusion of duplicates and repeated tests, the unadjusted positivity rate was 10.9% in 2012.

**“*Sentinella* study”**

**Summary of a study on consultations due to acute gastroenteritis in the Swiss Sentinel Surveillance Network, *Sentinella***

**Introduction**

It is estimated that 0.3 to 1.4 episodes of acute gastroenteritis per person-year occur in the general population in European countries [2-7]. In Switzerland, the burden of disease at primary care level is not known. We assessed the frequency of consultations, signs and symptoms, physicians’ diagnostic and treatment approaches, course of disease and inability to work in a study within the Swiss Sentinel Surveillance Network.

**Methods**

The Swiss Sentinel Surveillance Network consists of 150-250 general practitioners, internists and paediatricians participating on a voluntary basis. During the Sentinella-year 2014 (28.12.2013 – 26.12.2014), physicians participating in Sentinella reported all cases consulting due to acute gastroenteritis. They provided basic information on cases such as sex, age, stool testing and hospitalisation (both yes/no) on a weekly questionnaire. For a subsample of cases, physicians completed a supplementary questionnaire including more detailed information on treatment, stool diagnostics, inability to work and selected risk exposures.

**Selected preliminary results**

Stool testing was performed among 11% (420/3794) of patients as reported on weekly questionnaires.

Supplementary Table S1: Consultation and treatment characteristics of different patient groups with acute gastroenteritis. Preliminary results from the Swiss Sentinel Surveillance Network, Sentinella, 2014

|  | Patients without stool diagnostic | Patients with campylobacter-negative stool diagnostic | Patients with campylobacter-positive stool diagnostic |
| --- | --- | --- | --- |
| Corresponding to patient management model | A | B | C |
| Number of consultations (average) | 1.2 | 1.7 | 1.9 |
| Number of drug classes prescribed (of probiotics, antiemetics & antidiarrhoeals; average) | 1.1 | 1.0 | 0.9 |
| Antibiotics prescribed | 5% | 29% | 70% |

The two most frequently prescribed antibiotics in laboratory-confirmed campylobacteriosis patients were quinolones (24/47 responses) and macrolides (10/47 responses).

Supplementary Table S2: Unit cost prices used for calculation of health care costs for the four different patient management models

| Item | Points / cost weight | Point value / base rate (in €^a^) | Costs (in € ^a^) | Source |
| --- | --- | --- | --- | --- |
| **Physician / consultation costs** |  |  |  |  |
| Consultation |  |  |  |  |
| First 5 minutes | 17.76 | 0.7138 | 12.68 | [8, 9] |
| Each additional 5 minutes | 17.76 | 0.7138 | 12.68 | [8, 9] |
| Last 5 minutes | 8.88 | 0.7138 | 6.34 | [8, 9] |
| Telephone consultation |  |  |  |  |
| First 5 minutes | 17.76 | 0.7138 | 12.68 | [8, 9] |
| Each additional 5 minutes | 17.76 | 0.7138 | 12.68 | [8, 9] |
| Last 5 minutes | 8.88 | 0.7138 | 6.34 | [8, 9] |
| Physician service in absence of patient (incl. reviewing patient file), each 5 minutes | 17.76 | 0.7138 | 12.68 | [8, 9] |
| Taking blood sample | 8.19 | 0.7138 | 5.85 | [8, 9] |
| **Laboratory costs** |  |  |  |  |
| Stool culture (*Campylobacter*, *Salmonella*, *Shigella*), negative | 78 | 0.83 | 64.74 | [10] |
| Stool culture (*Campylobacter*, *Salmonella*, *Shigella*), positive | 155 | 0.83 | 128.65 | [10] |
| Haemogram (through automated method): erythrocytes, leucocytes, haemoglobin, haematocrit, thrombocytes, and 5 or more subpopulations of leucocytes | 12 | 0.83 | 9.96 | [10] |
| C-reactive protein (CRP), quantitative | 10 | 0.83 | 8.30 | [10] |
| **Medication / pharmacy costs** |  |  |  |  |
| Pharmacy fee: check of purchase |  |  | 2.70 | [11] |
| Pharmacy fee: check of prescription |  |  | 3.57 | [11] |
| Antibiotic^b^ |  |  | 24.90 | [12] |
| Medication (Probiotic, antiemetic or antidiarrhoeal)^c^ |  |  | 10.79 | [12] |
| **Hospital costs** |  |  |  |  |
| Hospital stay: DRG G67B | 0.573 | 8250.20 | 4727.36 | [13, 14] |
| ^a^ Average exchange rate for 2012 was used to convert Swiss francs to Euros: 1 CHF = 0.83€ [15] | | | | |
| ^b^ Average price for an antibiotic was calculated by (1) extracting all drugs with active agent erythromycin or ciprofloxacin, antibiotics which are recommended for treatment of campylobacteriosis according to Schweiger *et al.* [16] from the list of pharmaceutical specialties [12], (2) selecting drugs suitable for the recommended treatment of 2x500mg for 5 days [16] (hence, smallest packaging size with at least 10 tablets with 500mg of active agent), (3) calculating the average cost per active agent, and (4) calculating the average cost over the two active agents by using the ratio of erythromycin *vs.* ciprofloxacin as observed in the Sentinella study (one third erythromycin, two thirds ciprofloxacin) | | | | |
| ^c^ Average price for one drug (either probiotic, antiemetic or antidiarrhoeal) was calculated by (1) extracting all drugs with active agent *Enterococcus faecium SF68*, *Saccharomyces boulardii*, domperidone or loperamide from the list of pharmaceutical specialties [12], (2) selecting the smallest packaging size for each drug/brand, (3) calculating the average cost per active agent, and (4) calculating the average costs over all active agents. | | | | |

Supplementary Table S3 Comparison of costs estimated for patient management model C and actual costs of campylobacteriosis patients treated at the Swiss TPH Travel Clinic by type of provider (in Euro).

|  | **Patient management model C**  **consultation with  pos. stool culture** | | **Patient invoice** | |
| --- | --- | --- | --- | --- |
|  | **Minimal  scenario** | **Extended  scenario** | **Median** | **(Range)** |
| Physician | 63 | 82 | 153 | (20 – 455) |
| Laboratory excluding test for *Campylobacter* | 18 | 18 | 167 | (22 – 526) |
| Stool test for *Campylobacter* with positive result | 129 | 129 | 129 |  |
| Medication | 11 | 42 | 0^a^ | (0 - 47) |
| **Total** | **221** | **271** | **464** | **(179 – 1033)** |
| ^a^ Medications can only be delivered directly by physicians in case of emergency in the canton of Basel-Stadt, Switzerland, where our patients have been treated. In non-emergency cases, the physician prescribes the medication for purchase in a pharmacy. In the latter case, costs are not on the invoice from the physician. | | | | |


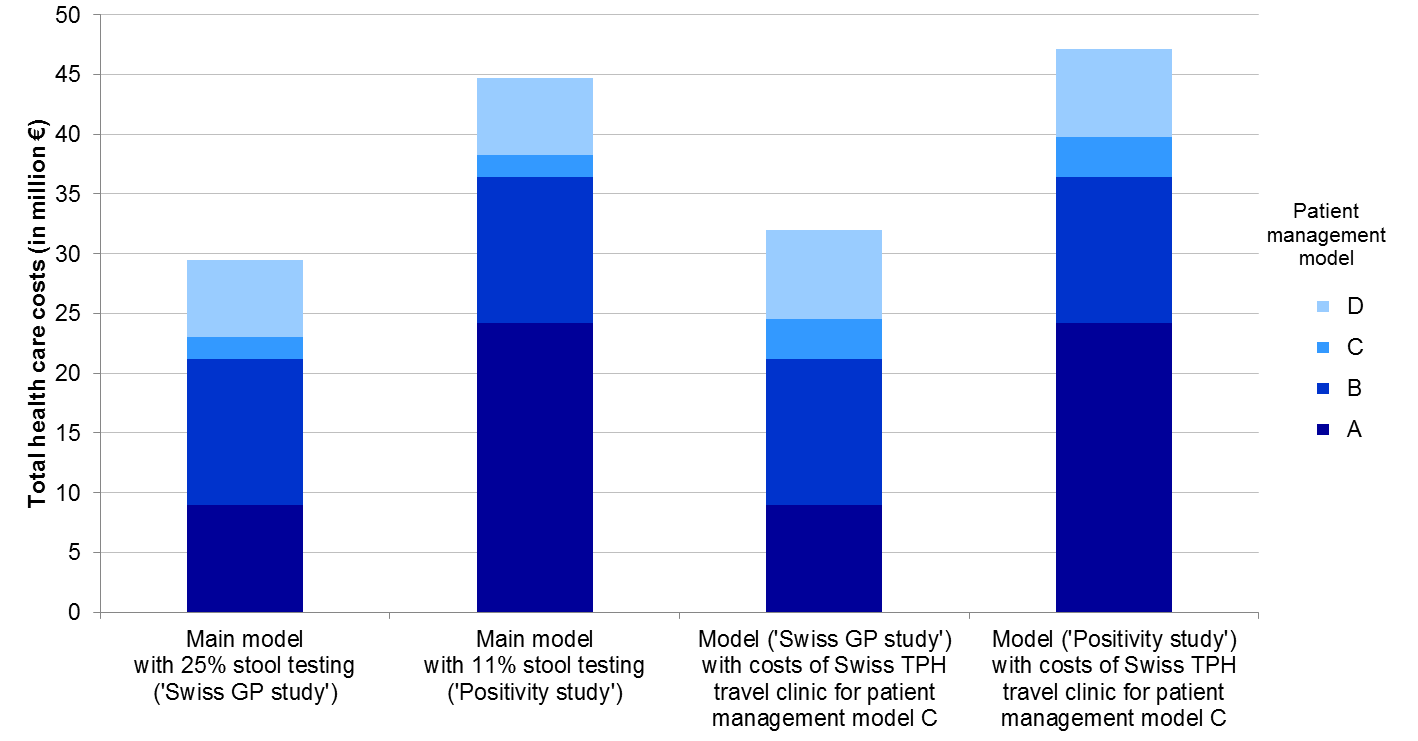


Supplementary Figure S1 Total health care costs for acute gastroenteritis and campylobacteriosis in Switzerland, by patient management model and under different model assumptions


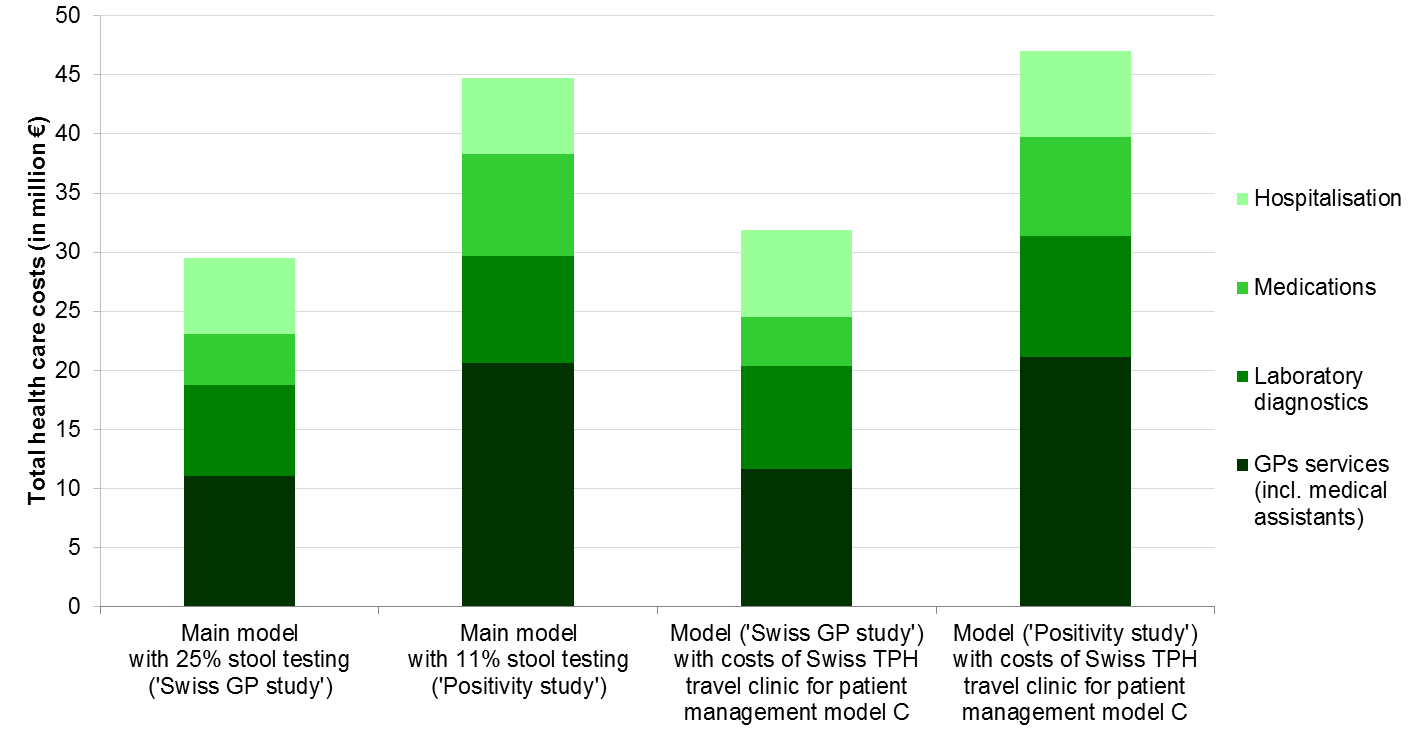


Supplementary Figure S2 Total health care costs for acute gastroenteritis and campylobacteriosis in Switzerland, by type of provider and under different model assumptions

**References**

(1) **Schmutz C*, et al.*** Inverse trends of *Campylobacter* and *Salmonella* in Swiss surveillance data, 1988-2013. *Eurosurveillance* 2016; **21**: pii=30130.

(2) **Tam CC*, et al.*** Longitudinal study of infectious intestinal disease in the UK (IID2 study): incidence in the community and presenting to general practice. *Gut* 2012; **61**, 69-77.

(3) **Wilking H*, et al.*** Acute gastrointestinal illness in adults in Germany: a population-based telephone survey. *Epidemiology and Infection* 2013; **141**, 2365-2375.

(4) **Doorduyn Y, Van Pelt W, Havelaar AH.** The burden of infectious intestinal disease (IID) in the community: a survey of self-reported IID in The Netherlands. *Epidemiology and Infection* 2012; **140**, 1185-1192.

(5) **Scavia G*, et al.*** The burden of self-reported acute gastrointestinal illness in Italy: a retrospective survey, 2008-2009. *Epidemiology and Infection* 2012; **140**, 1193-1206.

(6) **Kuusi M*, et al.*** Incidence of gastroenteritis in Norway - a population-based survey. *Epidemiology and Infection* 2003; **131**, 591-597.

(7) **Muller L, Korsgaard H, Ethelberg S.** Burden of acute gastrointestinal illness in Denmark 2009: a population-based telephone survey. *Epidemiology and Infection* 2012; **140**, 290-298.

(8) **NewIndex AG.** Kantonale Taxpunktwerte (http://www.newindex.ch/Taxpunktwerte-100). Accessed 20 April 2016

(9) **TARMED.** TARMED Tarif version 1.08.0000 of 1 June 2012 (http://www.tarmedsuisse.ch/pdf-tarifbrowser.html). Accessed 20 April 2016

(10) **Eidgenössisches Departement des Innern [Federal Department of Home Affairs].** Analysenliste vom 1. Januar 2012. Appendix 3 of "Verordnung des EDI vom 29. September 1995 über Leistungen in der obligatorischen Krankenpflegeversicherung (Krankenpflege-Leistungsverordnung, KLV) SR 832.112.31". Bern: 2012. Available from http://www.bag.admin.ch/al.

(11) **Anon.** Tarifvertrag (LOA IV) zwischen dem Schweizerischen Apothekerverband (pharmaSuisse) und santésuisse - Die Schweizer Krankenversicherer (santésuisse). Endversion vom 06.03.2009. [Agreement on tariffs between the Swiss organisation of pharmacies (pharmaSuisse) and santésuisse - the Swiss health insurers (santésuisse). Final version from 06.03.2009]. Available from http://www.pharmasuisse.org/data/Oeffentlich/de/Themen/Tarifvertrag_LOA-IV_def_d_09-03-6.pdf.

(12) **Bundesamt für Statistik.** Spezialitätenliste (SL) as per 1 January 2012 (www.sl.bag.admin.ch). Accessed 24 April 2015

(13) **SwissDRG AG**. Fallpauschalen in Schweizer Spitälern. In. Bern: SwissDRG AG, 2015: 8.

(14) **Gesundheits- und Fürsorgedirektion des Kantons Bern.** Übersicht stationäre Spitaltarife 2012 Kanton Bern (http://www.gef.be.ch/gef/de/index/gesundheit/gesundheit/spitalversorgung/spitaeler/superprovisorischetarife.assetref/dam/documents/GEF/SPA/de/Spitalversorgung/Tarife/Tarif%C3%BCbersicht_2012_d_141016.pdf). Accessed 06 November 2015

(15) **European Central Bank.** Euro foreign exchange reference rates (http://www.ecb.europa.eu/stats/exchange/eurofxref/html/index.en.html). Accessed 15 April 2016

(16) **Schweiger A, Markwalder K, Vogt M.** Infektiöse Diarrhoe: Epidemiologie, Klinik und Diagnostik. *Swiss Medical Forum* 2005; **5**, 714-723.
